# Effect of benthic boundary layer transport on the productivity of Mono Lake, California

**DOI:** 10.1186/1746-1448-4-11

**Published:** 2008-08-19

**Authors:** Louise C Bruce, Robert Jellison, Jörg Imberger, John M Melack

**Affiliations:** 1 Centre for Water Research, University of Western Australia, 35 Stirling Highway, Crawley, Western Australia, 6009, Australia; 2 Marine Science Institute, University of California, Santa Barbara, California, 93106-6150, USA; 3 Bren School of Environmental Science and Management, and Department of Ecology, Evolution and Marine Biology, University of California, Santa Barbara, California, 93106-9610, USA

## Abstract

The significance of the transport of nutrient-rich hypolimnetic water via the benthic boundary layer (BBL) to the productivity of Mono Lake was studied using a coupled hydrodynamic and ecological model validated against field data. The coupled model enabled us to differentiate between the role of biotic components and hydrodynamic forcing on the internal recycling of nutrients necessary to sustain primary productivity. A 4-year period (1991–1994) was simulated in which recycled nutrients from zooplankton excretion and bacterially-mediated mineralization exceeded sediment fluxes as the dominant source for primary productivity. Model outputs indicated that BBL transport was responsible for a 53% increase in the flux of hypolimnetic ammonium to the photic zone during stratification with an increase in primary production of 6% and secondary production of 5%. Although the estimated impact of BBL transport on the productivity of Mono Lake was not large, significant nutrient fluxes were simulated during periods when BBL transport was most active.

## Background

The transport of nutrient-rich water from benthic to pelagic regions has been linked to increased levels of primary productivity in stratified lakes [1-3]. Ostrovsky *et al*.. (1996) suggest that seiche activity in the boundary layer of Lake Kinneret sustained a vertical flux between the hypolimnetic and epilimnetic waters enhancing biological productivity in the lake. MacIntyre *et al*. (1999) calculated the upward fluxes of ammonium across the nutricline in Mono Lake and suggested nearshore boundary fluxes could be the dominant pathway supplying ammonium to the deep chlorophyll maxima. Eckert *et al*. (2002) used microstructure measurements of temperature, oxygen and hydrogen sulphide in Lake Kinneret to conclude that following the onset of stratification, the flux of benthic nutrients to the water column controls primary productivity. In this study we have defined BBL transport as that which occurs in the layer bordering the sediments of a lake [4,5] alternatively referred to as the bottom boundary layer [6].

The development of basin-scale internal waves arising from wind-induced energy are responsible for large scale water motions and most of the turbulence caused by these large-scale motions occurs in the BBL [7,8]. In order to differentiate between boundary and internal modes of vertical transport Yeates and Imberger (2004) parameterized the split between mixing in the internal and benthic boundary layer (BBL) using values of Lake number, L_N _[9] and Burger number, B_N _[10]. The L_N _is a measure of the amplitude of basin-scale internal waves in response to surface wind forcing, and B_N _describes waves that evolve from simple seiches [4]. Simulations performed on a number of monomictic lakes indicated that fluxes through the BBL were dominant during strong wind events occurring during period of stratification [4].

A number of studies, aimed at identifying sources and sinks of nutrients in the photic zone have focused on bacterial mineralization [11], regeneration through planktonic organisms [11-13], nitrogen fixation [14], hypolimnetic flux and inflows and outflows [15]. Although the occurrence of BBL transport and its potential impact on primary productivity has been examined, the upward mixing of nutrient-rich hypolimnetic waters via the BBL and the consequent effect on lake-wide ecological processes deserves further analysis.

Mono Lake is a nitrogen-limited saline lake with a relatively simple food web [16] and is subjected to wind-driven boundary-layer mixing events [1]. Yeates and Imberger (2004) simulated a BBL thickness in Mono Lake of 10–15 m during a sequence of strong wind event suggesting an active role in the development of the thermal structure of the lake. These features make it well-suited for examining the role of BBL-supplied nutrients and the influence of these nutrients on the seasonal plankton dynamics and overall productivity of the lake.

The objective of the present study is to investigate the role of BBL transport in the supply of nutrients to the photic zone and its consequent impact on the lake's ecology. A coupled hydrodynamic and ecological model was used to quantify nitrogen biogeochemistry during a 4-yr period from 1991–1994 when the lake mixed to the bottom during the winter. Initially, we calibrated the model parameters and processes to ensure an acceptable representation of the field data. The simulated output was then used to calculate the sources and sinks of nitrogen to the photic zone. A comparison could then be made between the roles of recycled and external sources on the primary and secondary productivity in the lake. To enable quantification of the significance of BBL transport for ecological processes, a series of simulations were run in which this mechanism was switched off allowing a comparison between lake behavior with and without BBL transport.

## Study Site

**Figure 1:**
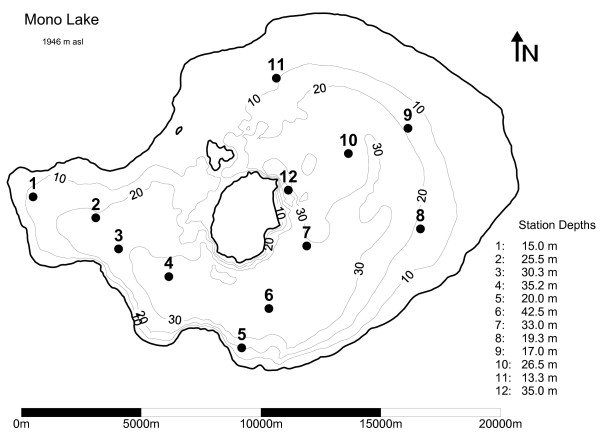
**Mono Lake**. Bathymetric map of Mono Lake showing sampling stations. Depth contours are in meters.

Mono Lake (38°N: 119°W) is a large saline lake with a salinity of 85–92 g kg^-1^, a maximum depth 45 m, mean depth 17 m and surface area approximately 160 km^2 ^(Fig. 1). The lake was monomictic during the period studied (1991–1994), and vertically mixed in winter (December to February) with thermal stratification beginning in early spring and persisting through autumn [17]. At other times following large runoff years, the lake experienced multi-year periods of chemical stratification (i.e., meromixis; 1982–1988, Jellison and Melack 1993b; 1995–2003, Jellison unpublished data). The present study examines four monomictic years (1991–94) to assess the effects of BBL on nutrient cycling and productivity during stratified and holomictic periods.

**Figure 2:**
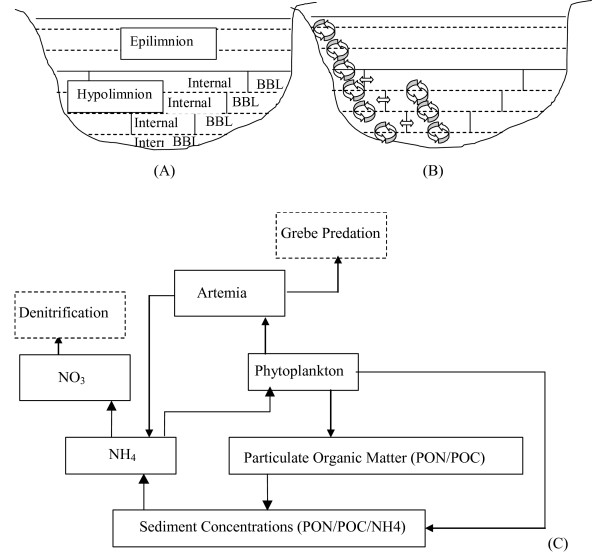
**Model schematic**. Schematic representation of (A) the model layer structure (B) internal and boundary layer mixing in the physical model DYRESM. (BBL: benthic boundary layer; Internal: internal cells; BC: benthic boundary layer cells) and (C) the carbon and nitrogen fluxes represented in the ecological model, CAEDYM. Dotted lines indicate that these variables are not included in model.

The planktonic community of Mono Lake has few species as is typical of hypersaline waters. The phytoplankton is dominated by a newly described picoplanktonic (2–3 μm) green alga, *Picocystis salinarum *Lewin (Lewin *et al*., 2000), and several bacillarophytes, mainly *Nitzschia *spp. (20–30 μm) (Lovejoy & Dana, 1977; Mason, 1967). A brine shrimp, *Artemia monica *Verill, is the only macrozooplankter (Lenz, 1980; Lenz, 1984). While pelagic ciliates and rotifers may also be present at times (Mason, 1967; Jellison et al. 2001), they contribute a negligible amount to the total zooplankton biomass.

There is a strong seasonal pattern in the nutrient and plankton dynamics of Mono Lake [18]. The seasonal patterns are driven by biotic and abiotic forces affecting productivity via bottom-up and top-down controls. Water temperatures of the surface mixed-layer ranged from 2–5°C in winter to 12–22°C in summer. Seasonal stratification and high productivity result in anoxic conditions in the hypolimnion where ammonium accumulates. The flux of this ammonium to the photic zone is limited until winter overturn mixes the whole lake providing nutrients for a pronounced spring algal bloom. Daily primary productivity rates are relatively high (Jellison and Melack 1993a).

The lake's only macrozooplankter, *A. monica*, produces over-wintering cysts that lie dormant on the bottom during the winter and hatch during early spring (February-April) [19]. *A. monica *biomass usually peaks in the late spring, remains high during the summer and gradually declines during the autumn as food is scarce and temperatures decline. The spring growth of *A. monica *biomass is associated with a simultaneous decline in phytoplankton biomass due to grazing and rise in surface concentrations of ammonium from zooplankton excretion. Phytoplankton biomass remains low during the summer and only increases toward the end of the year when grazing pressure is reduced [20].

As phosphorus concentrations are always high (>400 μM; Jellison et al. 1993), nitrogen limits primary production in the photic zone (Jellison & Melack 1993a, 2001). Nitrogen inputs from inflowing streams and planktonic nitrogen fixation are very low relative to internal fluxes where the main sources are from sediment release in the hypolimnion, phytoplankton and zooplankton excretion, and bacterial mineralization of particulate detrital organic nitrogen. Peak concentrations in the photic zone are observed at the breakdown of stratification as nutrient-rich hypolimnetic waters become entrained into the epilimnion. Towards the end of the mixed period and onset of stratification ammonium levels are generally low. When the zooplankton become abundant in late spring, grazing reduces phytoplankton biomass and internal phytoplankton nitrogen is converted to ammonium via the zooplankton grazing and excretion. Zooplankton excretion and reduced ammonium uptake due to low phytoplankton biomass results in an increase in epilimnetic ammonium concentrations.

## Results

### Nutrient concentrations, phytoplankton and zooplankton biomass

**Figure 3:**
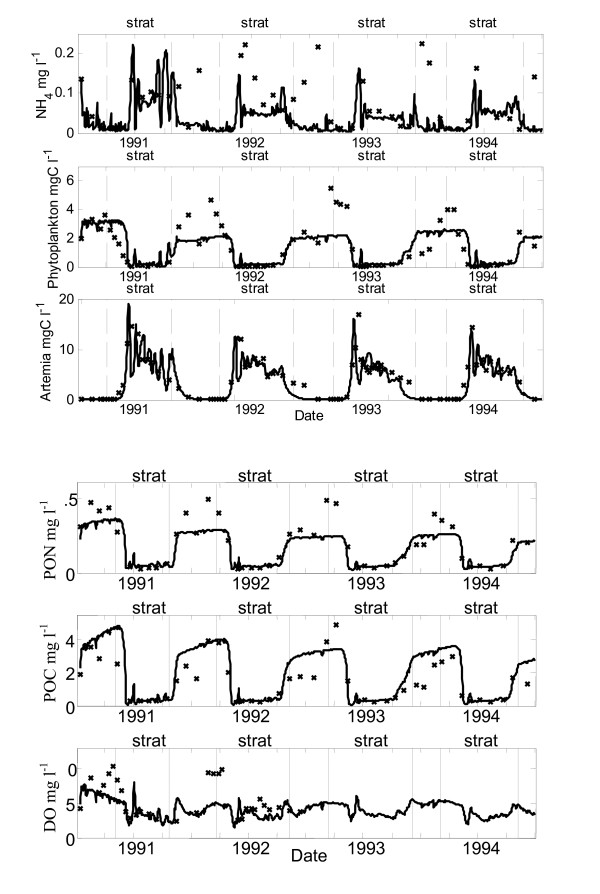
**Model simulations**. Comparison of model simulation results (lines) and field data (crosses) for Mono Lake from 1991 to 1994 for 9-m depth integrated averages of ammonium (NH_4_), total phytoplankton carbon (phytoplankton), total organic nitrogen (TPON), total organic carbon (TPOC) and dissolved oxygen (DO), vertical net tows of *Artemia monica *(Artemia).

The seasonal ammonium pattern of low winter concentrations and high summer values is reproduced by the model (Fig. 3). Similarly, peak concentrations of ammonium apparent in the observed data coinciding with the arrival of *A. monica *in the spring are matched in magnitude and timing by the model results. At the breakdown of stratification, the model simulated reduced ammonium concentrations corresponding to increased phytoplankton biomass (Fig. 3). However, the isolated high spikes in ammonium concentration observed in the field data during full circulation were generally not captured by the model (Fig. 3).

The simulated values of phytoplankton biomass follow the low summer concentrations, timing and slope of the autumn recovery and spring decline observed in the field data (Fig. 3). However, the elevated values of phytoplankton biomass observed in the field at the end of the mixing periods (early 1992, 1993 and 1994) are not captured by the model (Fig. 3).

The particulate organic nitrogen (PON) and particulate organic carbon (POC) data observed in the field closely followed that of the phytoplankton, with elevated values during the winter in the absence of grazing and low values during the summer months. These patterns were captured by the model, although elevated levels of PON were underestimated by the model during periods of full circulation (Fig. 3). However, elevated levels of POC were generally captured by the model which suggests that the model overestimated the detrital component of the particulate carbon pool (Fig. 3).

The timing and slope of the early spring peak in *A. monica *biomass observed in the field was matched in the simulated results across the four year simulation period (Fig. 3).

Simulated concentrations of dissolved oxygen are similar to those measured in the field during stratified periods (Fig. 3). The model, however, under predicted concentrations in late spring for both 1991 and 1992.

### Productivity and nitrogen fluxes

Primary productivity in Mono Lake has been estimated using a numerical interpolative model incorporating photosynthetic uptake rates and measured vertical attenuation of PAR [21]. During the non-meromictic conditions of 1989 and 1990, Jellison and Melack (1993a) estimated an average daily productivity of 1.6 g C m^-2 ^d^-1^. This matches the value simulated by DYRESM-CAEDYM for the 1991–1994 monomictic period. During periods of stratification an average daily productivity of 1.7 g C m^-2 ^d^-1 ^was simulated and 1.3 g C m^-2 ^d^-1 ^during periods of full circulation.

Average rates of lake-wide nitrogen deposition measured in 1986 and 1987 ranged from approximately 5.9 Mg N d^-1 ^(ca. 2.5 mmol m^-2 ^d^-1^) during the summer to 2.7 Mg N d^-1 ^(ca. 1.2 mmol m^-2 ^d^-1^) during the winter (Jellison *et al*. 1993). These rates are similar to those simulated by the model, i.e., 3.7 Mg N d^-1 ^and 2.1 Mg N d^-1 ^averaged during periods of stratification and full circulation, respectively. Areal average lake-wide nitrogen fluxes from the sediments were calculated by the model as 12.5 Mg N m^-2 ^d^-1 ^and 6.2 Mg N m^-2 ^d^-1 ^averaged during period of stratification and full circulation, respectively. Jellison *et al*. (1993) estimated the rate of ammonia release from the sediments based on sediment cores collected in 1988 as 58–162 Mg N m^-2 ^d^-1 ^(ca. 3.6–10.1 mmol m^-2 ^d^-1^). Although greater than those predicted by the model these estimates were derived under anoxic conditions so should be taken as an upper estimate.

### Measures of model performance

The calculated values of normalized mean absolute error, correlation coefficient and slope are presented in Table 3 for each of the main state variables over the full simulation period from 1991 to 1994 and compared to the calibration period from 1991 to 1992. Calculations of correlation coefficients are all equal to or greater than 0.8 with the exception of ammonium and dissolved oxygen.

### Sensitivity analysis

The five parameters that displayed the greatest sensitivity to annual estimates of lake-wide nitrogen fluxes were: (1) release rate of NH_4 _from sediments (S_dNH4_); (2) the fraction of zooplankton grazing excreted (f_ex_); (3) background attenuation coefficient (K_d_); (4) internal nitrogen to carbon ratio of the phytoplankton (IN_con_) and (5) the fraction of zooplankton grazing egested (f_eg_). The optimal parameter value (determined by the model best fit), and the upper and lower bounds used to determine alternative parameter sets are listed in Table 4.

### Nitrogen budget

**Figure 4:**
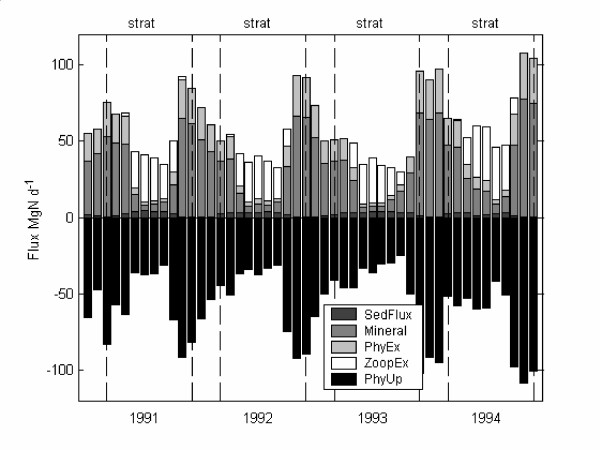
**Nitrogen fluxes**. Nitrogen fluxes (Mg N day^-1^) for total phytoplankton uptake (PhyUp) against sediment flux (SedFlux), mineralization of PON (Mineral), phytoplankton excretion (PhyEx), and zooplankton excretion (ZoopEx). Corresponding periods of stratification and full circulation are demarked by dashed lines.

Five major nitrogen fluxes were extracted from the model to compare the various component of the nitrogen budget (Fig. 4). These fluxes were: (1) phytoplankton uptake, (2) sediment to water exchange, (3) bacterially mediated mineralization, (4) phytoplankton excretion, and (5) zooplankton excretion. The model results are expressed as mass flux per day with respect to the whole lake, with phytoplankton uptake as a negative flux (sink) and the other four terms as positive fluxes (source) (Fig. 4). The results indicate that mineralization of particulate nitrogen made the greatest contribution to phytoplankton uptake in the winter and zooplankton excretion during the summer (Fig. 4). Sediment-released nitrogen fluxes are comparatively low, although significant in making up the difference between phytoplankton uptake and excretion (Fig. 4).

### Boundary layer mixing

**Figure 5:**
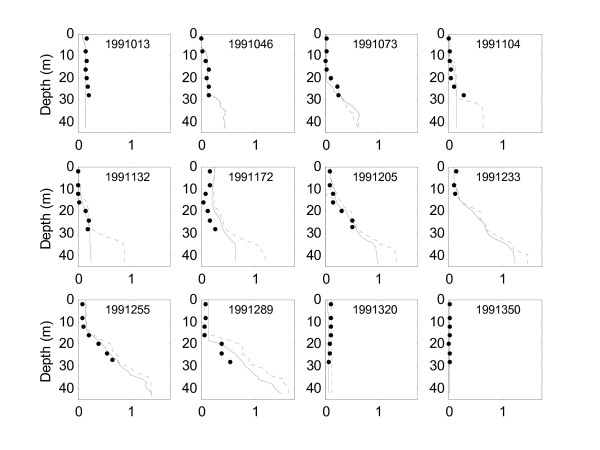
**BBL ammonium transport**. Comparison of NH_4 _(g m^-3^) depth profiles for the scenarios of BBL transport activated (solid line) and absent (dotted line) and field data (solid dots) for selected dates from 1991.

**Figure 6:**
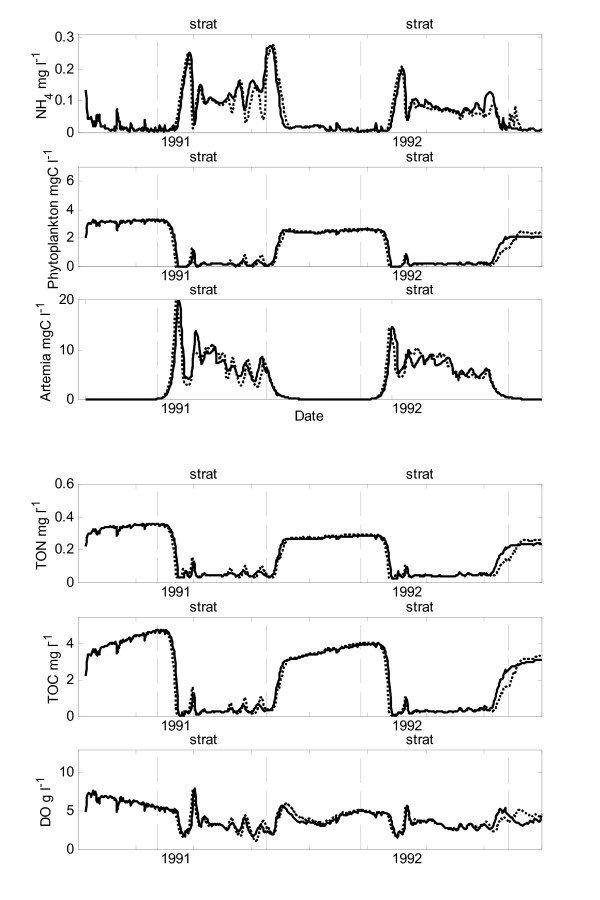
**Effect of BBL transport**. Comparison of model simulation results with BBL transport activated (solid lines) and absent (dotted lines) from 1991 to 1992 for 9-m depth integrated averages of ammonium (NH_4_), total phytoplankton carbon (phytoplankton), organic nitrogen (PON), organic carbon (POC) and dissolved oxygen (DO) and vertical net tows of *Artemia monica *(Artemia).

In the absence of BBL transport a greater buildup of ammonium in the hypolimnion was simulated, the difference being greatest in the early part of the stratified period (Fig. 5). However, the difference in the epilimnion is not so pronounced. Similarly, the simulated results of the 9 m depth averaged concentrations of ammonium, PON, POC, dissolved oxygen and phytoplankton and *A. monica *biomass indicated little difference between the alternative scenarios of BBL mixing (Fig. 6).

Calculations based on model output indicate that for 1991 to 1994 BBL transport was responsible for a 53% increase in upwards flux of ammonium across the thermocline during periods of stratification. For the corresponding periods, the simulated increase in primary production was calculated as 6% and secondary production as 5%. The model results, averaged over periods of autumn and winter mixing for the 4 years of simulation, indicated a reduction in upward ammonium flux of 28% when the BBL transport was active. This corresponded with a simulated decrease in primary production of 7% and negligible increase in secondary production of 1% for the same periods. The estimated net increase for 1991–1992 in ammonium flux across the thermocline due to BBL transport was 9%, primary productivity was 2% and secondary productivity was 3%.

**Figure 7:**
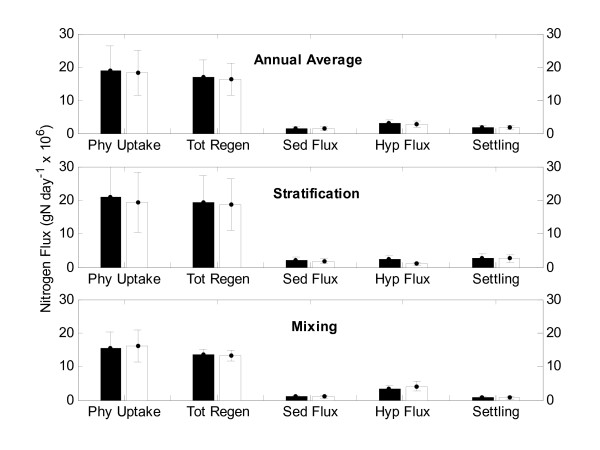
**Lakewide nitrogen fluxes**. Comparison of lake-wide nitrogen fluxes for phytoplankton uptake (Phy Uptake), total regenerated sources (Tot Regen), sediment flux (Sed Flux), flux across the thermocline (Hyp Flux) and settling of particulate nitrogen (Settling) averaged annually, during the stratified periods and during the mixed periods from 1991 to 1994 from the boundary mixing on (black bars) and off (white bars) scenarios. Error bars indicate one standard deviation from mean.

To place the differences in upward ammonium flux due to BBL transport in the context of the nitrogen cycle, the five major nitrogen fluxes were compared for both scenarios (Fig. 7). Almost no differences were found in the rates of regenerated nutrients, sediment flux and settling when BBL transport is inactive. Model results indicate that when the BBL transport was active ammonium flux across the thermocline accounts for 11% of the nitrogen sources to the photic zone during stratified periods. This compares to 5% when BBL transport is inactive.

## Discussion

Several aspects of the modeling require further examination. The step temperature function used to represent the process responsible for the hatching and initial growth of over-wintering *A. monica *cysts simulated well the timing and slope of the early spring peak in *A. monica *biomass. However, experiments have demonstrated that increases in salinity can influence the hatching process [22]. It is anticipated, therefore, that an additional salinity factor would be required before the model could be used to predict *A. monica *dynamics under alternative salinities. Although simulated mid-summer concentrations of *A. monica *compare favorably with those observed in the field, the autumn decline was difficult to simulate well (Fig. 3). The model included three processes responsible for decreases in biomass during this period; limited grazing at low temperatures, end of life mortality and grebe predation. Improved understanding of the combination of triggers responsible for the autumn decline in *A. monica *will aid in the model representation of these processes. Alternatively a cohort model such as that proposed by [23] may be required to accurately represent the autumn decline.

Differences between measures of fit comparing the calibration and validation periods are small. Although the ecological dynamics of the model during the validation period are similar to that of the calibration period, this result is an indication of model stability. However, this stability only relates to the representation of the interactions between the main processes responsible for determining the ecological patterns observed in the lake over the four years studied. Comparison to measures of fit for other lake ecosystem models is difficult as quantitative measures are rarely given. However, our overall measurement of NMAE compare favorably to Ross *et al*. (1994) (0.65) and Bruce *et al*. (2006) (0.52).

Since we defined sensitivity in relation to estimates of lake-wide nitrogen fluxes, it follows that the parameters showing the most sensitivity are related to the nitrogen cycle. Since the inflow of nitrogen into the lake is negligible, it follows that for Mono Lake, sediment release is a critical source of nitrogen to the water column. Similarly both the fractions of zooplankton grazing that goes into either egestion (the bulk of which is deposited into sediments and thus lost from the photic zone) or excretion (providing nitrogen in a form for primary production) have a direct effect on the proportion of phytoplankton nitrogen that is recycled. The ratio of phytoplankton internal nitrogen to carbon controls both the uptake of inorganic nitrogen by phytoplankton and the flux of nitrogen recycled via the zooplankton grazing and excretion pathway. Background attenuation influences nitrogen fluxes indirectly by controlling the amount of light available for primary productivity.

Model results indicated that during the summer stratified periods the N demand by phytoplankton in the surface to 9-m of Mono Lake is predominantly met by zooplankton excretion, phytoplankton leakage of dissolved organics, and bacterially mediated mineralization. Of these, the model predicted that the dominant source was zooplankton excretion. Since zooplankton biomass was well represented by the model including timing and magnitude of the initial peak, it follows that during these peaks the model has the closest fit to the ammonium data. Midwinter spikes in ammonium during period of reduced phytoplankton biomass were not reproduced in the model output. The model simulated almost constant phytoplankton biomass during the winter months that is inconsistent with the field data. From this we would conclude that the processes of phytoplankton ammonium uptake and release are not well represented by the model during winter conditions of high algal biomass and light-limitation. One of the limitations of this study was the assumption (for simplicity) of a constant internal phytoplankton C:N ratio. It is anticipated that modeling the internal nitrogen as a dynamic variable would improve the simulation of the phytoplankton-ammonium interactions particularly during periods of full circulation.

This study employed optimization techniques to determine a series of parameter sets to best represent field data as described by the processes included in the current model formulation. Some field data were better represented than others and misrepresentation of field data by the simulation will serve to direct improvements in future model generations. Although the modeled fluxes sometimes over or underestimated the measured concentrations, the general seasonal patterns were captured by the simulations and thus used to provide insight into the processes that determine the ecosystem dynamics of Mono Lake.

Bruce *et al*. (2006) in their study of the role of zooplankton in the nutrient cycles of Lake Kinneret, Israel, found zooplankton excretion to be the dominant source of dissolved nitrogen during winter overturn and sediment release the dominant source during summer stratification. For Mono Lake we also found that zooplankton excretion was most influential in the summer stratified period. Although the simulated rate of ammonium flux from the sediments was higher in Lake Kinneret [24], the main reason for finding sediment-released nutrients relatively less important in Mono Lake is due to two-fold higher rates of primary productivity and greater recycling due to zooplankton excretion in Mono Lake.

MacIntyre and Jellison (2001) suggested that transport of nutrient-rich hypolimnetic water via the BBL layer is responsible for increased ammonium flux across the thermocline and consequential increase in productivity. By comparing the simulation results from the two scenarios we found that, although the increase in upward ammonium flux across the thermocline during the stratified periods of 1991–1994 due to BBL transport was 53% (± 4%), primary productivity for the same period increased only 6% (± 4%). Since the model suggested that 87% of the N demand by phytoplankton is met by regenerated sources, it is not unexpected that an increase in external supply has a limited impact. MacIntyre *et al*. (1999) highlighted the importance of the flux of BBL transported ammonium across the thermocline in sustaining primary productivity to the deep chlorophyll maxima. As a percentage of phytoplankton demand during the stratified periods the upward flux of ammonium across the thermocline was calculated as 12% with BBL on and 5% with BBL off. MacIntyre *et al*. (1999) reached a similar conclusion and, assuming that 5–10% of primary productivity occurs in the deep chlorophyll maximum during the summer, suggested that BBL may be the dominant mechanism supplying ammonium to the deep chlorophyll maximum.

As anticipated, during stratified periods simulation results indicate that BBL transport leads to an increase in ammonium transport across the thermocline and concomitant increase in primary productivity. However, this pattern was reversed during periods of mixing. A greater build up of ammonium in the hypolimnion occurred during stratification in the case where BBL transport is absent (Fig. 6). Although in the absence of BBL transport, less flux was available in the photic zone during stratification, at overturn a greater mass of ammonium led to greater upwards flux of ammonium and concomitant increase in primary productivity. As a result, on an annual average, primary productivity was similar under both scenarios.

Our model results have illustrated the importance of timing of BBL transport and its subsequent effect on primary and secondary productivity. The model used in this study did not include algorithms to represent differences in generations using a stage-structured zooplankton model. Inclusion of a stage structured model might enable us to determine whether the timing of BBL transport events and concomitant increases in primary productivity effect the timing and magnitude of successive generations of *A. monica *in Mono Lake.

**Figure 8:**
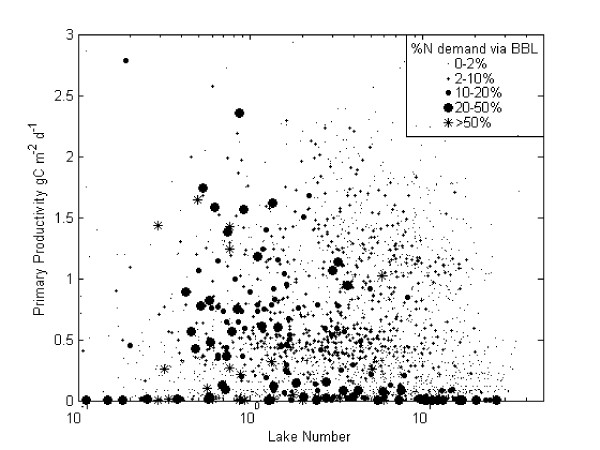
**Ammonium flux versus lake number**. Flux of ammonium transported via the BBL as a fraction of lake-wide vertical fluxes (closed circles) versus daily average values of Lake Number (L_N_) and Burger Number (B_N_).

It is apparent that one of the reasons the transport of ammonium via the BBL does not have a significant impact on the productivity of Mono Lake is that sediment released nutrients are not a major component of the nutrient cycle. Model results have confirmed previous studies indicating that productivity is predominantly sustained by recycled nutrients (Jellison *et al*.. 1993). Furthermore, simulated estimates of BBL volume from 1991–1994 revealed that, on average, the benthic boundary layer comprised only 1% by volume and stored only 1% of the lake-wide nitrogen mass. To investigate the potential importance of BBL transport for shallower lakes where the volume of BBL may be larger in proportion to the lake volume we ran three additional simulations. The same Mono Lake input files for 1991–1994 were used, lowering the surface level of the lake to simulate initial depths of 35 m, 30 m and 22 m. Combining the results of these simulations we plotted the flux of ammonium transported via the BBL as a fraction of N demand by phytoplankton against daily average values of Lake Number (L_N_) and primary productivity (Fig. 8). Simulated output indicated that the fraction of N demand met by hypolimnetic nutrients transported upwards in the BBL rarely exceeds 50% and only when primary productivity is minimal or for L_N _close to 1. The L_N _is inversely proportional to the thermocline height and is both a measure of the energy available at the thermocline from wind induced surface stress and the volumetric importance of the hypolimnion [9].

For Lake Kinneret, estimates of monthly primary productivity fall between 0.5 and 1.7 g C m^-2 ^d^-1 ^(Bruce *et al*. 2006). Mean daily values of L_N _estimated for Lake Kinneret range from 10^-2 ^to 100 with a period of low L_N _associated with strong wind events (Yeates and Imberger 2004). Given these ranges, it is predicted that the importance of BBL transport in Lake Kinneret may be greater than the 6% predicted for Mono Lake. In Lake Geneva, a study investigating the effect of internal waves on basin exchange indicated up to 40% of the hypolimnetic volume was exchanged following episodes of strong winds [25]. Primary productivity in Lake Geneva is relatively high [26]. For Lake Constance, estimates of L_N _during stratification are relatively high (Yeates and Imberger 2004) and productivity is less than 1 g C m^-2 ^d^-1 ^[27] suggesting that for Lake Constance the transport of nutrients through the BBL may be less important to overall lake productivity but potentially significant during episodic events associated with low L_N_.

The results of this study have indicated that the relative importance of BBL transport as a source of nutrients sustaining productivity in the photic zone is determined by productivity and morphology. Future studies will be focused on comparing the effect of BBL transport on the ecology of other lakes. By differentiating between physical and ecological process we will be able to determine what limnological features alter the importance of BBL transport.

## Methods

### Model description

The model used in this study is a modified version of the Computational Aquatic Ecosystem Dynamics Model (CAEDYM) [28,29] coupled to the Dynamic Reservoir Model (DYRESM) [4]. In DYRESM the lake is represented as a series of homogeneous horizontal layers of variable thickness [4]; as inflows and outflows enter or leave the lake, the affected layers expand or contract, respectively, and those above move up or down to accommodate the volume change. Mass, including that of the ecological state variables, is adjusted conservatively each time layers expand, contract, merge or are affected by inflows and outflows. The main processes modelled in DYRESM are surface heat, mass and momentum transfers, mixed layer dynamics, hypolimnetic mixing, benthic boundary layer mixing, inflows and outflows.

Local meteorological data are used to determine heating due to short-wave radiation and surface heat fluxes due to evaporation, sensible heat, long-wave radiation and wind stress. The surface wind field introduces both momentum and turbulent kinetic energy to the surface layer contributing to vertical mixing. In addition to surface layer mixing, DYRESM includes algorithms that account for internal mixing (encompassing the effects of internal wave energized shear mixing) and benthic boundary layer (BBL) mixing (determined by the turbulent kinetic energy budget and parameterized by Lake number and the Burger number). The total volume of water (*F*_*i*_^*T*^) exchanged by deep water mixing and transport processes for layer *i *is determined by the following equation:

(1) $$F_{i}^{T}=\frac{200N_{i}^{2}A_{i}K_{M}∆t}{L_{N}N_{\max}^{2}\left(\frac{\delta_{i}+\delta_{i+1}}{2}\right)}$$

where *N*^2 ^is the buoyancy frequency, *A *is the layer area (m^2^), *K*_*M *_is the molecular diffusion coefficient for heat, Δ*t *is the time step (seconds), *L*_*N *_is the Lake number and *δ*_*i *_the layer thickness of layer *i *(m) [4]. In this way mass transfer is enabled from hypolimnetic layers to the thermocline region internally and via the BBL. A recent modification of the DYRESM code is the separation of these mass transfers described in detail by Yeates and Imberger (2004). The Lagrangian layers have been separated into internal and BBL cells so volume exchange occurring beneath the surface mixing layer can be separated into that associated with internal mixing (between internal cells) and that associated with benthic boundary layer mixing (between BBL cells; Fig. 2A&B). The volume exchange is partitioned into BBL (*F*_*i*_^*B*^) and internal (*F*_*i*_^*I*^) using the following equation:

(2) $$\begin{array}{ccc} F_{i}^{I}=\{\begin{array}{ll} \frac{F_{i}^{T}\tanh\left(B_{N}\right)\left(L_{N}-1\right)}{L_{N}} & L_{N}>1\\ 0 & otherwise \end{array} & and & F_{i}^{B}=F_{i}^{T}-F_{i}^{I} \end{array}$$

where B_N _is the Burger number [4].

The ecological model CAEDYM was set up in the form of an 'N-P-Z' (nutrients-phytoplankton-zooplankton) model (Fig. 2C) with resolution to the level of individual species or groups of species [24]. In the present study it is used to simulate phosphorus and nitrogen in both particulate and dissolved inorganic forms (POP and PO_4_, PON, NO_3_, NH_4_), dissolved oxygen (DO), particulate organic carbon (POC), dissolved organic carbon (DOC), one phytoplankton group representing *Picocystis *sp. and one zooplankton group representing *A. monica*. A series of ordinary differential equations is used in CAEDYM to describe changes in concentrations of nutrients, detritus, dissolved oxygen, phytoplankton and zooplankton as a function of environmental forcing and ecological interactions for each cell represented by DYRESM (Table 1). The variables of irradiance, temperature, salinity and density are also passed to CAEDYM at each 1-hr time step and used in equations to determine rates of change of biomass and chemical constituents for each of the ecological state variables. The two models CAEDYM and DYRESM share the same layer structure including the division of hypolimnetic layers into two cells, BBL and internal. The BBL cells are considered adjacent to sediment cells so that sediment exchange of nutrients and dissolved oxygen occurs to and from these cells. The physical transfer of ecological variables between adjacent cells due to various mixing processes is accounted for in DYRESM. Further details of the structure of CAEDYM are given in Robson and Hamilton (2004) and Romero *et al*. (2004).

**Table 1:** Model process equations. Equations used to describe the processes included in the ecological model CAEDYM

∂Z_i_/∂t = [G_i_A_i_*f*(Z)_i_*f*_1_(T)(1-f_ex_-f_eg_) - (R_i_+M_i_)*f*_2_(T) - Pred_i_]Z_i_
= (assimilation - excretion - egestion) - (respiration + mortality) - predation
∂P/∂t = [P_max,j_*f*_1_(T)min(*f*(I),*f*(P),*f*(N)) - (R_j_)*f*_2_(T) - Pred_j_]P_j _± S_j_
= photosynthetic uptake - (respiration + excretion + mortality) - predation ± settling
∂POC/∂t = Σ[G_i_*f*(Z)_i_*f*_1_(T)_i_((1-A_i_) + A_i_f_eg_) + M_i_*f*_2_(T)_i_]Z_i _+ Σ[R_j_(1-f_res_)(1-f_DOM_)*f*_2_(T)]P_j _- Pred_POC_POC - R_POC_*f*(DO)*f*_1_(T)POC ± S_POM_
= (unassimilated zooplankton food + zooplankton egestion + zooplankton mortality) + phytoplankton mortality - zooplankton predation - POC decomposition ± settling
∂DOC/∂t = Σ[R_j_(1-f_res_)f_DOM_*f*_2_(T)]P_j _+ R_POC_*f*_POC_(POC)*f *(DO)*f*_2_(T)POC - R_DOC_*f *(DO)*f*_2_(T)DOC
= phytoplankton excretion + POC decomposition - DOC mineralisation
∂POP/∂t = Σ[G_i_*f*(Z)_i_*f*_1_(T)_i_((1-A_i_) + A_i_f_eg_) + M_i_*f*_2_(T)_i_]IP_Zi_Z_i _+ Σ[R_j_(1-f_res_)(1-f_DOM_)*f*_2_(T)]IP_j _- Pred_POC_POP - R_POP_*f*(DO)*f*_1_(T)POP ± S_POM_
= (unassimilated zooplankton food + zooplankton egestion + zooplankton mortality) + phytoplankton mortality - zooplankton predation - POP decomposition ± settling
∂DOP/∂t = Σ[R_j_(1-f_res_)f_DOM_*f*_2_(T)]IP_j _+ Σ[A_i_f_ex_G_i_*f*(Z)_i_*f*_1_(T)_i_]IP_Zi_Z_i _+ R_POP_*f *(DO)*f*_1_(T)POP - R_DOP_*f *(DO)*f*_1_(T)DOP
= phytoplankton release + zooplankton excretion + POP decomposition - DOP mineralisation
∂PO4/∂t = R_DOP_*f *(DO)*f*_2_(T)DOP - Σ[UN_max,j_*f*_1_(T)_j_*f*(IP)_j_*f*(P)_j_]P_j _+ S_dPO4_*f*(DO)*f*_2_(T)LA/LV
= DOP mineralisation - phytoplankton uptake + PO4 sediment flux
∂PON/∂t = Σ[G_i_*f*(Z)_i_*f*_1_(T)_i_((1-A_i_) + A_i_f_eg_) + M_i_*f*_2_(T)_i_]IN_Zi_Z_i _+ Σ[R_j_(1-f_res_)(1-f_DOM_)*f*_2_(T)]IN_j _- Pred_PON_PON - R_PON_*f*(DO)*f*_1_(T)PON ± S_POM_
= (unassimilated zooplankton food + zooplankton egestion + zooplankton mortality) + phytoplankton mortality - zooplankton predation - PON decomposition ± settling
∂DON/∂t = Σ[R_j_(1-f_res_)f_DOM_*f*_2_(T)]IN_j _+ Σ[A_i_f_ex_G_i_*f*(Z)_i_*f*_1_(T)_i_]IN_Zi_Z_i _+ R_PON_*f*(DO)*f*_2_(T)PON - R_DON_*f*(DO)*f*_2_(T)DON
= phytoplankton release + zooplankton excretion + PON decomposition - DON mineralisation
∂NH4/∂t = R_DON_*f*(DO)*f*_1_(T)DON - Σ[UN_max,j_P_N_*f*_1_(T)_j_*f*(IN)_j_*f*(N)_j_]P_j _- R_NO_*f*(DO)*f*_2_(T)NH4 + S_dNH4_*f*(DO)*f*_2_(T)LA/LV
= PON mineralisation - phytoplankton uptake - nitrification + NH4 sediment flux
∂NO3/∂t = R_NO_*f*(DO)*f*_2_(T)NH4 - R_N2_*f*(DO)*f*_2_(T)NO3 - Σ[UN_max,j_(1-P_N_)*f*_1_(T)_j_*f*(IN)_j_*f*(N)_j_]P_j_
= nitrification - denitrification - phytoplankton uptake
∂DO/∂t = k_O2_(DO_atm - DO) + Σ[P_max,j_*f*_1_(T)_j_min(*f*(I),*f*(P),*f*(N)) - R_j_*f*_2_(T)_j_]P_j_Y_O2:C _Σ[R_i_*f*_2_(T)_i_]Z_i_Y_O2:C _- R_DOC_*f *(DO)*f*_1_(T)DOCY_O2:C _- R_NO_*f *(DO)*f*_2_(T)NH4 - S_dO2_*f*(DO)*f*_2_(T)LA/LV
= atmospheric flux + (phytoplankton oxygen production - phytoplankton respiratory consumption) - zooplankton respiratory consumption - utilisation of oxygen in mineralisation of DOM - utilisation of oxygen in nitrification - sediment oxygen demand.

Temperature functions
*f*_1_(T) = θ^T-20 ^- θ^k(T-a) ^+ b
where k, a and b are constants solved numerically to satisfy the following conditions:
*f*_1_(T) = 1; at T = Tsta
∂*f*_1_(T)/∂T = 0; at T = Topt
*f*_1_(T) = 0; at T = Tmax
*f*_2_(T) = θ^T-20^

Limitation equations
*f*(Z)_i_=(ΣP_j_+ΣZ_k_+POC)/(K_i_+ΣP_j_+ΣZ_k_+POC)
*f*(I)_j _= I/I_s _exp(1-I/I_s_)
f(IP)_j _= [IP_max_/(IP_max_-IP_min_)] [1-IP_min_/IP]
f(IN)_j _= [IN_max_/(IN_max_-IN_min_)] [1-IN_min_/IN]
f(DO) = DO/(K_DO_+DO)
f(P) = PO4/(K_PO4_+PO4)
f(N) = (NH4+NO3)/(K_N2_+NH4+NO3)
P_N _= (NH4 NO3)/[(NH4+K_N_)(NO3+K_N_)] + (NH4 K_N_)/[(NH4+K_N_)(NO3+K_N_)]

Settling
S_j _= (ws/Δz)P_j_
S_POM _= (g(ρ_POM _- ρ_w_)(D_POM_)^2^/18μ)/Δz)POM

Predation
Pred_i _= Σ(G_k_f(Z)_k_f_1_(T)_k_Z_k_PzZOO_k,i_)
Pred_j _= Σ(G_i_f(Z)_i_f_1_(T)_i_Z_i_PzPHY_i,j_)
*Abbreviations*: Z, zooplankton; P, phytoplankton; POC, particulate organic carbon; DOC, dissolved organic carbon; POP, particulate organic phosphorus; PO_4_, phosphate; PON, particulate organic nitrogen; NH_4_, ammonium; NO_3_, nitrate; POM, particulate organic matter (C, N or P); IP_zi_, zooplankton internal phosphorus; IN_zi_, zooplankton internal nitrogen; IP_j_, phytoplankton internal phosphorus; IN_j_, phytoplankton internal nitrogen; DO, dissolved oxygen; DOatm, concentration of oxygen in the atmosphere; LA, layer area; LV, layer volume; Δz, layer thickness; ρ_w_, density of water; μ, viscosity of water; k_O2_, oxygen transfer coefficient. *Subscripts*: i, zooplankton group; j, phytoplankton group; k, zooplankton predator group.

The major nutrient fluxes represented in CAEDYM are uptake of dissolved inorganic nutrients by phytoplankton, release of dissolved nutrients from phytoplankton excretion, grazing, egestion and excretion of nutrients by zooplankton, nitrification and denitrification of inorganic nitrogen, sedimentation of nutrients in particulate form, mineralization of organic nutrients and release of dissolved nutrients from sediments (Table 1).

Net change in carbon concentration of the phytoplankton at each model time step is calculated as the difference between the increment due to gross primary production and losses due to sedimentation, grazing by zooplankton, respiration, excretion and mortality. These terms are calculated using equations parameterized to represent the physiology of the main phytoplankton species. Losses due to grazing by zooplankton are calculated by multiplying the food assimilation rate for the zooplankton by a preference factor for phytoplankton over detrital POC.

Net zooplankton growth is calculated as a balance between food assimilation and losses from respiration, excretion, egestion, predation and mortality. Food assimilation is calculated as the product of the maximum potential rate of grazing, assimilation efficiency, and temperature and food concentration functions. A constant internal nutrient ratio is assumed and excretion of nutrients calculated to maintain this ratio at each time step. Advective movement of zooplankton is carried out in DYRESM.

Bacteria have not been directly simulated as they were not measured during the study period. However the nutrient pathways mediated by bacteria were included as mineralization of the particulate organic pools (POC, POP and PON). The POC, POP and PON pools available for zooplankton grazing include bacteria. Predation of zooplankton by grebes was included by an additional predation term for the months of August to November estimated from predation studies (Cooper *et al*. 1984).

The advantage of using a depth resolved model DYRESM linked to the ecological model CAEDYM is that we could explore the effect of transport and mixing between the epilimnion, metalimnion and hypolimnion on the ecological processes in the lake. Of most relevance to this study is the exchange of nutrient-rich hypolimnetic waters to the photic zone via the BBL and its consequential effect on the primary productivity. The separation of internal and BBL cells in the layered structure of the current DYRESM allowed us to differentiate between the transport of nutrients in the internal and BBL and to determine the relative importance of each process on the mixed layer ecological dynamics. The ecological model also returns the attenuation coefficient (as a function of the concentration of both phytoplankton and particulate organic matter) to the hydrodynamic model at each one-hour time step. This variable is used to determine the extent of light and heat penetration that in turn governs the deepening of the surface mixed layer and the timing of winter turnover. In this way the feedback on a sub-daily time scale between the ecological and physical models is instrumental in the application of the model to aid in understanding of the interaction of various lake processes.

### Field sampling and analytical analysis

Seasonal and year-to-year variations in the physical, chemical, and biotic environments were monitored fortnightly from March through October and monthly during November through January. Water temperature and conductivity were measured at nine buoyed, pelagic stations (2, 3, 4, 5, 6, 7, 8, 10 and 12) (Fig. 1). Profiles were taken with a high-precision, conductivity-temperature-depth profiler (CTD) (Seabird Electronics model Seacat 19) equipped with a submersible photosynthetically available radiation (PAR) (LiCor 191S), fluorescence (695 nm) (WETLabs WETStar miniature fluorometer), and transmissivity (660 nm) (WETlabs C-Star Transmissometer). Specific conductivity, salinity, and density were all calculated based on equations derived from measurements on Mono Lake brine [30]. Dissolved oxygen was measured at one centrally located station (Station 6) with a Yellow Springs Instruments temperature-oxygen meter (YSI, model 58) and probe (YSI, model 5739). The oxygen electrode was calibrated at least once each year against Miller titrations of Mono Lake water (Walker *et al*. 1970).

Ammonium and chlorophyll profiles were determined by sampling 7–10 discrete depths at two pelagic stations (2 and 7; Fig. 1), while *A. monica *abundance was determined via vertical net tows collected at 10 (1991–1992) or 20 (1993–1994) pelagic stations (Fig. 1). Chlorophyll *a *was also determined in the upper water column from samples collected with a 9-m integrating tube sampler at 5 pelagic stations (2, 6, 7, 10, 11; Fig. 1).

Nutrient and phytoplankton samples were immediately passed through a 120-μm net to remove all stages of *A. monica *and a sub-sample filtered through Gelman A/E glass fiber filters for analysis of nutrients (Jellison and Melack 1993a). Ammonium concentrations were measured with the indophenol blue method as described by Jellison *et al*. (1993). Nitrate and nitrite concentrations were measured but were always low (< 1 μM) [21,31] and thus not considered in this study. Phosphate concentrations are orders of magnitude greater than the half saturations constants for phytoplankton so were not considered in this study [21]. Phytoplankton chlorophyll *a *was determined by spectrophotometric analysis as described by Jellison and Melack (1993a). Conversion of chlorophyll to carbon units were made by assuming a C:Chl *a *ration of 50 (see Jellison & Melack 2001). Subsamples were filtered onto precombusted Gelman A/E filters for the determination of particulate organic carbon (POC) and nitrogen (PON). Duplicate carbon and nitrogen filters were acid fumed for 12 hours over concentrated HCl, and then dried at 40–50°C before determination by combustion in a Perkin-Elmer 240B elemental analyzer standardized with acetanilide. *A. monica *were collected using vertical net tows (120-μm mesh) to within 1-m of the bottom or well below the oxycline depending on stratification. *A. monica *biomass (dry weight) was estimated from stage-specific abundance, adult female length data, and weight-length relationship determined in the laboratory simulating in situ conditions of food and temperature [32]. Conversion from dry weight to carbon assumed 0.4 g C/g dry weight [33,34].

#### Model inputs

Model input files included data for initialization, meteorology, inflows and outflows. The initialization file was prepared from field data collected on 13 January 1991. On this day the temperature of the lake ranged from 2.5 to 3°C and the salinity from 88 to 88.5 g kg^-1^. Inflow data included the daily volume, temperature and salinity for two inflows, one representing total surface inflows (streams and direct runoff) and the other, hydrothermal springs. The volume of the hydrothermal springs was set at 3888 m^3 ^day^-1^, based on a ^3^He mass balance of Mono Lake [35]. The surface inflows were calculated based on a water mass balance using measured values of water depth and evaporation calculated by DYRESM. As ammonium, phytoplankton and zooplankton concentrations are negligible in the inflows, they were set to zero (Jellison and Melack 2001). Meteorological input data included hourly short- and long-wave radiation, air temperature, vapor pressure, wind speed and precipitation [36]. Air temperature, vapor pressure (converted from relative humidity), wind speed and precipitation were collected at a meteorological station located on Paoha, a central island (Fig. 1). Radiation data were collected from a meteorological station located approximately 7 km from the southwest shore of the lake (Fig. 1).

The physical parameters used to simulate the hydrodynamics of Mono Lake were either physical constants or ones fixed according to the dimensions of the lake [4].

The formulation of CAEDYM used here to describe the ecological variables and processes required 57 parameters determined by several methods (Table 2). Most phytoplankton parameters were derived from experimental analysis on the predominant phytoplankton species of Mono Lake [21,37]. The zooplankton parameters were determined where available from experiments conducted on *A. monica *or alternative *Artemia *species (Table 2). If parameters were not available, a series of model runs were performed to calibrate the simulation results against field data, maintaining parameter values within the bounds of literature values measured in other lakes.

**Table 2:** Model parameters. Parameters used in CAEDYM to simulate ecological variables in Mono Lake.

*General*				
Parameter	Description	Units	Assigned value	Values from field/lit

K_d_	Background extinction coefficient	m^-1^	0.35	0.29–0.34^a^
Source	^a ^Calculated from unpub data on in-situ light measurements			
				
*Phytoplankton*				

Parameter	Description	Units	Assigned values:	Values from field/literature
P_max_	Maximum potential growth rate	d^-1^	5.96	7.2^a^

I_K_	Parameter for initial slope of PI curve	μEm^-2^s^-1^	25	25^b^
Kep	Specific attenuation coefficient	m^2 ^g C^-1^	0.008	0.008^c^
K_P_	Half saturation constant for phosphorus uptake	mg L^-1^	0.001	Low value as not P limited
K_N_	Half saturation constant for nitrogen uptake	mg L^-1^	0.0573	Calibrated
IN_con_	Constant internal N ratio	mg N (mg C)^-1^	0.0926	0.17^d^
IP_con_	Constant internal P ratio	mg P (mg C)^-1^	0.026	0.048^d^
θ_j_	Temperature multiplier for growth		1.06	1.07^e^
T_sta_	Standard temperature	°C	19	
T_opt_	Optimum temperature	°C	22	
T_max_	Maximum temperature	°C	39.5	
R_j_	Metabolic loss rate coefficient	d^-1^	0.302	Calibrated
θ_R_	Temperature multiplier for metabolic loss		1.05	Calibrated
f_res_	Fraction of respiration relative to total metabolic loss		0.693	Calibrated
f_DOM_	Fraction of metabolic loss rate that goes to DOM		0.291	Calibrated
ws	Settling velocity	m d^-1^	0.008	0.04–0.013^f^
Sources	^a^Jellison and Melack 1993a, based on maximum value of carbon uptake measured from lake samples 1983–1990 assuming 50 g C g Chl *a*^-1^ ^b ^Jellison and Melack 1993a, based on minimum value of I_K _measured from lake samples 1983–1990. ^c^Jellison and Melack 1993a. ^d^Jellison and Melack 2001, estimated from seston ratios during the summer period from monomictic years 1991–1995 1984 ^e^Jellison and Melack 1993a, based on Q10 of 1.95. ^f^Jellison *et al*.. 1993.			
				
*Zooplankton*				

Parameter	Description	Units	Assigned values:	Values from field/literature

G_i_	Grazing rate	g C m^-3 ^(g C m^-3^)^-1 ^d^-1^	1.12	1.26^a^
A_zi_	Grazing efficiency	-	1.0	Close to 1 as filter feeders
R_i_	Respiration rate coefficient	d^-1^	0.113	0.035–0.1^b^
M_i_	Mortality rate coefficient	d^-1^	0.0107	0.0033^c ^0.0262^d^
f_eg_	Fecal pellet fraction of grazing	d^-1^	0.096	Kfz+kez = 0.36–0.68^e^
f_ex_	Excretion fraction of grazing	d^-1^	0.49	
DOmz	Minimum DO tolerance	mg L^-1^	0.0	0–1.2^f^
θ_i_	Temperature multiplier for growth		1.055	1.22^g^
Tmin	Minimum temperature	Deg C	6	6.8–9.0^h^
θ_Ri_	Respiration temperature dependence		1.10	
K_i_	Half saturation constant for grazing	g C m^-3^	1.12	2.96^i^
IN_zi_	Internal ratio of nitrogen to carbon.	g N g C^-1^	0.208	0.197/0.218^j^
IP_zi_	Internal ratio of phosphorus to carbon	g P g C^-1^	0.02	0.0135^k^
PzPHY	Preference of zooplankton for phytoplankton		0.8	
PzPOC	Preference of zooplankton for POC		0.2	
Sources	^a ^[33,44] (*Artemia fransiscana *optimal food 11 days old) ^b^[33,44] (*Artemia fransiscana *range of food 11 days old) ^c^Jellison *et al*.. 1993 (based on survival rate over 30 days) ^d^Dana and Lenz 1986 (based on survival rate over 26 days) ^e^Evjemo *et al*.. 2000, *Artemia fransiscana*. ^f^DO concentration at depth of deep Chl *a *maxima (unpub data). ^g^Jellison *et al*.. 1993 (best fit to temperature function used in model) ^h ^Jellison unpub data 1991–1994. (based on temperature at which total biomass < 0.01 before Spring growth) ^i ^Evjemo and Olsen 1999 (*Artemia fransiscana *11 days old, 26–28°C, Holling Type II) ^j ^Jellison unpub data (Females/Males) ^k ^[45]			
				
*Dissolved Oxygen and Nutrients*				

Parameter	Description	Units	Assigned values	Values from field/literature

S_dDO_	DO sediment exchange rate	g m^-2^d^-1^	0.053	
K_DO_sed_	Half saturation constant for DO sediment flux	mg O L^-1^	0.537	
K_DO_POM_	Half saturation constant for dependence of POM/DOM decomposition on DO	mg O L^-1^	1.46	
fanB	Aerobic/anaerobic factor	-	0.357	
θ_POM_	Temperature multiplier	-	1.03	1.02–1.14^a^
R_POC_	Mineralisation rate for POC to DOC	d^-1^	0.12	
R_POP_	Mineralisation rate for POP to DOP	d^-1^	0.1	0.01–0.1^a^
R_PON_	Mineralisation rate for PON to DON	d^-1^	0.4	0.01–0.03^a^
D_POM_	Diameter of POM particles	m	0.000009	
ρ_POM_	Density of POM particles	kg m^-3^	1109	
KePOC	Specific light attenuation coefficient for POC	m^2 ^g^-1^	0.00943	
R_DOC_	Mineralisation rate for DOC	d^-1^	1	Set to 1 to eliminate DOP pool for simplicity
R_DOP_	Mineralisation rate for DOP to PO4	d^-1^	1	Set to 1 to eliminate DOP pool for simplicity
R_DOP_	Mineralisation rate for DOP to PO4	d^-1^	1	Set to 1 to eliminate DOP pool for simplicity
R_DON_	Mineralisation rate for DON to NH4	d^-1^	1	onset to 1 to eliminate DON pool for simplicity.
KeDOC	Specific light attenuation coefficient of DOC	m^2 ^g^-1^	0.001	
R_N2_	Denitrification rate coefficient	d^-1^	0.000864	0.1^a^
θ_N2_	Temperature multiplier for denitrification	-	1.08	1.045^a^
K_N2_	Half saturation constant for denitrification dependence on oxygen	mg N L^-1^	1.75	
R_NO_	Nitrification rate coefficient	d^-1^	0.00553	0.1–0.2^a^
θ_NO_	Temperature multiplier for nitrification	-	1.08	1.08^a^
K_NO_	Half saturation constant for nitrification dependence on oxygen	mg O L^-1^	0.5	
θ_sed_	Temperature multiplier for sediment nutrient fluxes	-	1.05	
S_dNH4_	Release rate of NH4 from sediments	g m^-2 ^d^-1^	0.0712	0.054–0.18^b^
K_DO_SdNH4_	Controls sediment release of NH4 via oxygen – Half saturation constant for sediment NH4 release dependence on DO	g m^-3^	0.565	
Sources	^a^Jorgensen and Bendoricchio 2001 ^b ^Jellison *et al*.. 1993			

**Table 3:** Normalised mean absolute error.

Variable	NMAE	SD/Mean	r^2^	Slope
NH_4_	0.58 (0.56)	0.88 (0.72)	0.37 (0.59)	0.29 (0.61)
Phytoplankton	0.45 (0.44)	1.15 (1.04)	0.79 (0.85)	0.61 (0.80)
*Artemia monica*	0.30 (0.30)	0.85 (0.95)	0.79 (0.83)	0.80 (0.87)
TPON	0.35 (0.34)	0.83 (0.82)	0.90 (0.94)	0.52 (0.57)
TPOC	0.43 (0.42)	0.91 (0.88)	0.86 (0.92)	1.02 (1.15)
Dissolved oxygen	0.31 (0.31)	0.48 (0.48)	0.64 (0.64)	0.32 (0.32)
Average	0.40 (0.40)	0.85 (0.82)	0.72 (0.79)	0.59 (0.72)

Results of normalised mean absolute error (NMAE) calculations applied to compare simulated to field data for simulated years 1991–1994. The values in brackets represent the same calculations made over the 1991–1992 calibration period.

**Table 4:** Sensitivity analysis.

Parameter	Optimal	Lower bound	Upper bound	NMAE (lower bound)	NMAE (upper bound)
S_dNH4_	0.06	0.01	0.10	0.49	0.42
f_ex_	0.50	0.05	0.70	0.49	0.44
K_d_	0.30	0.35	0.25	0.41	0.43
IN_con_	0.09	0.07	0.22	0.47	0.49
f_eg_	0.16	0.05	0.20	0.47	0.42

The minimum and maximum values of the five most sensitive parameters and the corresponding results of normalised mean absolute error (NMAE) calculations applied to compare simulated to field data for simulated years 1991–1994. The values in brackets represent the same calculations made over the 1991–1992 calibration period.

A variety of quantifiable measures of model fit are described in Alewell and Manderscheid (1998). We choose the average absolute error normalized to the mean (NMAE):

(3) $$NMAE=\frac{\sum_{t=1}^{n}\left(\left|s_{t}-o_{t}\right|\right)}{n\bar{o}}$$

where *s*_*t *_is the simulated value at time *t*, *o*_*t *_is the observed value at time *t*, *ō *is the mean of the observed values over the simulation period and *n *is the number of observed values. *NMAE *is a measure of the absolute deviation of simulated values from observations, normalized to the mean; a value of zero indicates perfect agreement and greater than zero an average fraction of the discrepancy normalized to the mean. To compare the extent of variability within the observed data, we also calculated for each state variable, the standard deviation of observed data normalized to the mean over the simulation period (Table 3). In addition, the correlation coefficients and associated slope for the direct comparison of observed against simulated values for each state variable were calculated.

The period from 1991–1992 was used for initial parameter calibration. Comparisons of field and model data were made for six major model state variables: (1) ammonium (NH_4_); (2) particulate organic nitrogen (PON); (3) particulate organic carbon (POC); (4) dissolved oxygen (DO); (5) phytoplankton carbon and (6) zooplankton carbon. PON and POC refer to the sum of phytoplankton and detrital particulate nitrogen and carbon, respectively. Lake-wide averages of the surface to 9-m integrated concentrations of NH4, PON, POC, DO and phytoplankton carbon were compared. For zooplankton, lake-wide averaged biomass (g C m^-2^) as determined by vertical net tows were compared to vertically-integrated model output.

A manual calibration procedure was initially applied whereby individual parameters were adjusted and the model response observed. The particular features of the observed data that were used to adjust individual parameters were dependent on the parameter adjusted. For example, the minimum temperature for *A. monica *growth was adjusted to gain best representation of the timing of the spring zooplankton peak and the grazing rate adjusted to gain best representation of the magnitude of this peak. Individual parameters were adjusted in this way until an overall model average NMAE (calculated using the field data from the five variables listed above) of less than 0.5 was achieved.

Once a reasonable fit was achieved through trial-and-error, the local parameter space optima was determined by applying a Levenberg-Marquant (L-M) method of optimization [38] using a predefined Matlab^® ^function (The MathWorks Inc., Natick, MA). In this function, parameters were adjusted to optimize the sum of the NMAE values for the same five variables listed above. Additional bounds were placed on simulated values of primary productivity and nitrogen sedimentation to fall within the ranges of those estimated by Jellison *et al*. (1993). The years 1993–1994 were used for model validation.

Uncertainly in model predictions arises from different sources including those associated with process representation, parameter estimation, uncertainty in inputs and observed data [39-42]. While a full analysis of model uncertainty is beyond the scope of this paper, we made an estimate of the uncertainty associated with parameter estimation by comparing output from simulations using ten different parameter sets. Initially, the five most sensitive parameters to model output were established by the sensitivity analysis described below. We then determined alternative parameter sets by fixing the lower and upper bound of each parameter and then optimizing the remaining parameters via the L-M method described above until appropriate calibration was achieved. A benchmark NMAE value of 0.5 was selected so that calibration was deemed successful if the NMAE was less than 0.5 (Table 4). The lower and upper bounds for each sensitive parameter were determined by experimental or literature ranges. Model output from this suite of parameter sets was then used as an estimate of the relative uncertainty in model output. As the upper and lower bound of the five most sensitive parameters were used, this should provide a conservative estimate of the model uncertainty associated with parameter estimation.

To determine the five parameters most sensitive to model output, a sensitivity analysis was performed on each of the CAEDYM parameters listed in Table 2. Sensitivity coefficients (*s*_*ij*_) to assess the relative sensitivity of variable i to parameter j were calculated according to:

sij=Δcic¯iΔβjβ¯j

where Δ*c*_*j *_is the change in output variable *i *from the reference value *c*_*i *_and Δ*β*_*j *_is the change in parameter *j *from the reference value *β*_*j *_[43]. Because this study is concerned with the role of physical transport mechanisms on lake-wide nitrogen fluxes, we focused on the response of the five major nitrogen fluxes (phytoplankton uptake, sediment flux, zooplankton regeneration, settling and upward flux into surface mixed layer) to parameter manipulation. Each parameter was adjusted by ± 10% or by ± 0.01 in the case of the temperature multipliers. A time variant array of sensitivity parameters was calculated for each flux and then the average taken and used to rank the parameters according to sensitivity.

To enable us to quantify the significance of BBL transport on the ecological processes of the lake, a series of simulations were run in which this mechanism was switched off allowing a comparison between lake behavior with and without BBL transport. In DYRESM the lake-wide vertical fluxes are partitioned into internal and BBL contributions (see eq.36 in Yeates and Imberger (2004)). The BBL contribution was set to zero with all other parameters (physical and ecological) remaining the same. The simulated output of nitrogen fluxes and primary and secondary production were then analyzed and compared against base line output.

## List of abbreviations used

BBL: Benthic boundary layer; CAEDYM: Computational aquatic ecosystem dynamic model; DYRESM: Dynamic reservoir simulation model.

## Declaration of competing interests

The authors declare that they have no competing interests.

## Authors' contributions

All authors participated in the conception and design of the study. RJ collected data used in model validation while LCB conducted model simulations and drafted the initial manuscript. All authors contributed to revising and editing the manuscript, and have read and approved the final version.
